# The Ubiquitin Ligase Nedd4-1 Participates in Denervation-Induced Skeletal Muscle Atrophy in Mice

**DOI:** 10.1371/journal.pone.0046427

**Published:** 2012-10-26

**Authors:** Preena Nagpal, Pamela J. Plant, Judy Correa, Alexandra Bain, Michiko Takeda, Hiroshi Kawabe, Daniela Rotin, James R. Bain, Jane A. E. Batt

**Affiliations:** 1 Keenan Research Centre of the LiKaShing Knowledge Institute, St Michaels Hospital, Toronto, Ontario, Canada; 2 Clinical Science Division, Department of Medicine, University of Toronto, Toronto, Ontario, Canada; 3 Department of Molecular Neurobiology, Max-Planck-Institute of Experimental Medicine, Goettingen, Germany; 4 Program in Cell Biology, The Hospital for Sick Children, Toronto, Ontario, Canada; 5 Department of Surgery, McMaster University, Hamilton, Ontario, Canada; St. Jude Children's Research Hospital, United States of America

## Abstract

Skeletal muscle atrophy is a consequence of muscle inactivity resulting from denervation, unloading and immobility. It accompanies many chronic disease states and also occurs as a pathophysiologic consequence of normal aging. In all these conditions, ubiquitin-dependent proteolysis is a key regulator of the loss of muscle mass, and ubiquitin ligases confer specificity to this process by interacting with, and linking ubiquitin moieties to target substrates through protein∶protein interaction domains. Our previous work suggested that the ubiquitin-protein ligase Nedd4-1 is a potential mediator of skeletal muscle atrophy associated with inactivity (denervation, unloading and immobility). Here we generated a novel tool, the *Nedd4-1* skeletal muscle-specific knockout mouse (*myo^Cre^;Nedd4-1^flox/flox^*) and subjected it to a well validated model of denervation induced skeletal muscle atrophy. The absence of Nedd4-1 resulted in increased weights and cross-sectional area of type II fast twitch fibres of denervated gastrocnemius muscle compared with wild type littermates controls, at seven and fourteen days following tibial nerve transection. These effects are not mediated by the Nedd4-1 substrates MTMR4, FGFR1 and Notch-1. These results demonstrate that Nedd4-1 plays an important role in mediating denervation-induced skeletal muscle atrophy *in vivo*.

## Introduction

Skeletal muscle atrophy is a pathophysiologic consequence of disease progression in multiple chronic illnesses such as cancer, heart failure, HIV/AIDS and endstage pulmonary disorders. Loss of muscle mass also occurs with muscle inactivity due to denervation injury and immobility/unloading, and as a consequence of normal aging or starvation. In all illnesses, skeletal muscle atrophy impairs function, and enhances disease morbidity [1]–[5].

Ubiquitin-mediated proteolysis was identified as a critical regulator of skeletal muscle atrophy in several animal models of disease [6]–[8]. In the ubiquitination process, the co-ordinated action of several enzymes (E1, E2 and E3 ubiquitin ligases) covalently attaches a single or branched ubiquitin moiety to lysine residue(s) of the target substrate protein, which can serve as a marker for regulated cellular degradation of the protein by the 26S proteasome or the lysosome [9]. The E3 ligases are the key enzymes that confer specificity to the system by binding and linking ubiquitin moieties to the substrate, often through protein∶protein interaction domains [9]. There are two main families of E3 ligases; HECT (homologous to E6-AP carboxy-terminus) domain E3 ligases [10] and RING (Really interesting new gene) finger ligases [11], [12].

Two muscle specific ubiquitin ligases, the RING containing atrogin-1/MAFbx and MuRF1, are involved prominently in the development of skeletal muscle atrophy following metabolic insult [13], [14], as well as atrophy resulting from denervation and immobility/unloading [15]. Both atrogin-1 and MuRF1 knock-out mice are partially, but not fully, protected from the development of skeletal muscle atrophy. We, and others, have demonstrated increased expression of the HECT domain ubiquitin ligase Nedd4-1 (Neural precursor cell expressed developmentally down-regulated protein 4) in the muscle of rodents subjected to models of hind-limb unloading and denervation induced muscle atrophy [15], [16]. In contrast to atrogin-1 and MuRF1, which increase only in short term denervated muscle in rats, Nedd4-1 expression was sustained long term, suggesting that it plays a more encompassing role in the induction of skeletal muscle atrophy [15]. Koncarevic *et al*. have demonstrated that in rodents, the increase in Nedd4-1 occurs exclusively in muscle atrophy associated with muscle inactivity, such as denervation, disuse and unweighting [16]. We have also demonstrated that Nedd4-1 level is increased in the atrophic muscles of individuals with severe Chronic Obstructive Pulmonary Disease (COPD) [17]. We speculate that Nedd4-1, like its RING E3 counterparts, is a critical E3 ligase in the induction of inactivity induced muscle proteolysis, and that the absence of Nedd4-1 in skeletal muscle will spare its atrophy following insults associated with inactivity.

A second Nedd4 isoform, Nedd4-2, is expressed in human and mouse [18]–[20]. Despite their structural similarities, Nedd4-1 and Nedd4-2, for the most part, target distinct substrates and serve distinct functions [21]. Nedd4-1 is the predominant skeletal muscle isoform [19] and in *Drosophila* Nedd4-1 (dNedd4) was shown to regulate neuromuscular synaptogenesis [22]. Nedd4-1 germline deficient (complete null) mice are embryonic/perinatal lethal, exhibiting cardiac and vascular defects, and abnormalities of neurite development [23], [24]. Nedd4-1 null embryonic mice also demonstrate abnormalities in neuromuscular junction formation [25] and possibly a skeletal muscle phenotype, developmental muscle hypoplasia, although the relevance of this finding remains uncertain since the Nedd4-1 null embryos demonstrate a global reduction in body size and muscle size was not normalized to body size [25]. Others have reported the muscle developmental delay in Nedd4-1 null embryonic mice as part of a global developmental delay [26]. The role of Nedd4-1 in post-natal muscle is unknown.

Our goal here was to assess the role of Nedd4-1 in the development of denervation induced skeletal muscle atrophy *in vivo* using a genetic (mouse) model. To this end, we generated *Nedd4-1* skeletal muscle-specific knockout (*Nedd4-1* SMS-KO) mice, and subjected them to a well validated model of denervation induced muscle atrophy. We report the *Nedd4-1* SMS-KO mice demonstrate partial protection from muscle atrophy, exhibiting heavier denervated muscle weights and larger type II fibre cross sectional areas compared to their wild type littermates. These findings show that Nedd4-1 plays an important role in the regulation of denervation- induced skeletal muscle atrophy in animals.

## Materials and Methods

### Generation of *Nedd4-1* skeletal muscle-specific knockout mice

Mice with a floxed *Nedd4-1* allele (*Nedd4-1^flox/flox^*) reported in our previous study [23] were crossed with a mouse line expressing Cre-recombinase under the control of the mouse *myogenin* promoter and the skeletal muscle specific enhancer of the mouse *MEF2C* gene [*myo-cre* (*myo^Cre^*) mouse, generously supplied by Dr. Eric Olson, University of Texas, South Western] [27]. Both of these regulatory elements are active only in the skeletal muscle lineage from embryonic day (E) 8.5 to adulthood, thus allowing us to generate a mouse line in which Nedd4-1 was deleted specifically from skeletal muscle. Mice heterozygous for the floxed *Nedd4-1* allele and for the *myo-cre* transgene (*myo^Cre^;Nedd4-1^flox/+^*) were bred to create *Nedd4-1* SMS-KO (*myo^Cre^;Nedd4-1^flox/flox^*) mice and control littermates (*myo^Cre^;Nedd4-1^+/+^*, *Nedd4-1^+/+^* and *Nedd4-1^flox/flox^*) mice.

### Experimental denervation model and muscle preparation

The well validated gastrocnemius muscle denervation model [28] was employed and approved by the Research Ethics Board, Hamilton Health Sciences Corporation, McMaster University (AUP # 10-04-24). The study was carried out in strict accordance with the recommendations of the Canadian Council on Animal Care. The right tibial nerve was transected under inhalational Halothane anaesthesia completely denervating the gastrocnemius muscle in *Nedd4-1* SMS-KO mice and littermate *myo^Cre^;Nedd4-1^+/+^* control mice at the age of three to five months. The proximal portion of the tibial nerve was sutured to the superficial surface of the biceps femoris muscle to prevent errant re-innervation of the gastrocnemius muscle. The contralateral leg served as an internal control in each animal. This manipulation permits full weight bearing by the mouse on the both hind limbs during ambulation. Mice were maintained under conditions of routine care for 1 or 2 weeks post-nerve transection. Subsequently, the mice were sacrificed and the gastrocnemius muscles were harvested from the operated experimental limb and contralateral control limb. After rapid, atraumatic dissection and weighing, the muscle was split and fixed in 10% buffered formalin or snap frozen in liquid nitrogen. Fixed tissue was processed for immunohistochemistry and morphometric measurements. Frozen muscle was used for extraction of cellular protein.

### Western Blotting

Total protein was extracted by homogenizing (Polytron PT 1200E, Kinematica, Lucerne, Switzerland) the muscle in Muscle Lysis Buffer [5 mM Tris-HCl pH 8.0, 1 mM EDTA, 1 mM EGTA, 1 mM β-mercaptoethanol, 1% glycerol, PMSF (1 mM), leupeptin, aprotinin (10 ug/ml each)] for 3×30 sec. and homogenates were centrifuged at 1600×g for 10 min at 4°C. The supernatant was cleared by centrifuging further for 10 min at 4°C, 10,000×g. All fractions were quantified using the Pierce (Rockford, IL, USA) BCA Protein Assay Kit and normalized for equal loading for SDS-PAGE and Western blot analysis.

25 or 50 µg of muscle lysates were separated by SDS-PAGE, transferred to nitrocellulose, Ponceau-S stained and immunoblotted. The commercial primary antibodies used included anti-Nedd4-1 (BD-Biosciences, Franklin Lakes, NJ, USA, #611480), - Hypoxanthine-guanine-phosphoribosyltransferase (HPRT; Abcam, Cambridge, MA, USA #109021), -Pax-7 (Developmental Studies Hybridoma Bank, DSHB, University of Iowa, Iowa City, IA, USA), -Nedd4-2 (Cell Signalling Technologies, Danvers, MA, USA, #4013), -Fibroblast Growth Factor Receptor 1 (FGFR1; Abcam #824), -Notch-1 (Cell Signalling) and anti- Glyceraldehyde-3-phosphate dehydrogenase (GAPDH, Abcam, #8245) antibodies. Secondary antibodies were Horseradish peroxidase (HRP)-linked anti-rabbit, anti-mouse or anti-rat and protein bands detected with Biorad ECL (Biorad Laboratories, Hercules, CA, USA) or Immun Star Western C (Biorad Laboratories). Quantification of the chemiluminescent signal from Western blots was performed with Bio-Rad Fluor S Max Acquisition System (Biorad Laboratories) and Image Lab software (Biorad Laboratories).

The anti-MTMR4 (Myotubularin related protein 4) antibody was generated in our laboratory against a Glutathione transferase (GST)-fusion protein of the C terminal region of MTMR4 (RARELMSQQLKKPIATASS; aa 1172–1195, accession # CAQ52069), that was purified from bacteria with glutathione agarose beads, then subjected to thrombin cleavage to release the MTMR4 specific peptide. As the peptide molecular weight is only approx 3 kDa and we were unable to visualize the final product on a Coomassie stained gel, we obtained confirmation of peptide size with mass spectrometry analysis (data not shown). A polyclonal antibody was generated in rabbit following injection of the purified peptide. Specificity of the antibody was confirmed by assessing pre-immune and post-immune serum on Western blotting of HEK293 cell lysates untransfected and transfected with HA-tagged MTMR4 cDNA (data not shown).

### Immunohistochemistry and muscle morphometrics

Gastrocnemius muscles were fixed in 10% buffered formalin phosphate for 24 hours at room temperature, rinsed in ethanol, paraffin embedded and sectioned (10 µm thickness) on cross section. The sections were rehydrated in a series of xylene and ethanol washes and then endogenous peroxidases were quenched with a 30 min. incubation in 0.3% H_2_O_2_. Antigen retrieval was performed by microwaving the sections in 10 mM sodium citrate, pH 6.0, 3×5 min at 90% power (720 Watt oven). Sections were blocked with Protein Block – Serum Free (Dako/Agilent, Mississauga, Canada). Sections were rinsed and fibres were immunostained using either anti-skeletal muscle myosin, fast isoform antibody (MY-32, Sigma) specific to fast-twitch muscle fibres, or anti-Pax-7 (Pax 7, Developmental Studies Hybridoma Bank, University of Iowa, USA), a marker of muscle satellite cells/myoblasts, followed by biotinylated secondary antibody and streptavidin-HRP/DAB (Vectastain ABC Elite Peroxidase kit, Vector Laboratories Inc., Burlingame, CA, USA). For a negative control, the primary antibody was omitted during immunostaining. Hematoxylin was used as a counterstain. Cross sectional areas (CSA) of type I and type II fibres were determined using ImageJ software (NIH) by 3 independent reviewers blinded to mouse genotype. A minimum of 300 fibres per muscle were measured.

### Primary Satellite cell/myoblast Isolation and Culture

Primary myoblast cultures were generated as previously described [29]. Briefly, hindlimb muscles were dissected from *Nedd4-1* SMS-KO and littermate *myo^Cre^;Nedd4-1^+/+^* control mice, and incubated in Dulbecco's Modified Eagles Medium (Gibco BRL, Rockville, MD, USA) with 2% (w/v) collagenase II (Gibco BRL, Grand Island NY, USA) for 90 min at 37°C. Digested muscle was washed in growth media (GM; Hams F10 media with 20% Fetal Bovine Serum, 5 ng/mL basic fibroblast growth factor, 1% penicillin/streptomycin) and dissociated into single myofibres with repetitive trituration using a glass Pasteur pipette. To further isolate satellite cells, myofibres were washed 2 more times aggressively to remove adherent cells (satellite cells are situated beneath the basal lamina). Satellite cells were liberated by further digesting the myofibre fragments in 10 volumes of PBS, 0.5 U/mL dispase (Invitrogen, Life Technologies, Grand Island NY, USA) and 38 U/mL collagenase type II (US Biological, Swampscott, MA, USA) for 30 min at 37°C with agitation. Fetal bovine serum (1∶10 by volume) was added to digests to block further enzymatic reactions, followed by centrifugation at 500×g for 1 min to settle debris. The resulting supernatant was filtered through a 50 micron mesh, centrifuged at 1000×g for 5 min to pellet satellite cells. The cells were then re-suspended in GM and pre-plated onto a plastic tissue culture dish in GM for 40 min to remove any remaining fibroblasts (which adhere readily to plastic). Satellite cells were transferred and cultured in GM to glass cover slips in 6 well plates coated with lamin 5 ug/mL (Gibco, BRL) and 0.002% collagen (Sigma) and grown at 37°C in 5% CO2.

### Immunocytology

Myoblast cultures were fixed with 4% paraformaldehyde in PBS for 15 min, washed with PBS and paraformaldehyde was subsequently deactivated with 100 mM glycine in PBS for 10 min. Cells were washed, then permeabilized in buffer containing 0.1% Triton X-100 diluted in PBS for 20 min at room temperature (RT). Cells were washed with PBS and then blocked with the addition of 5% BSA in PBS for 1 hour at RT. Cells were labelled with rabbit anti-MyoD (Santa Cruz Biotechnologies, Santa Cruz CA, USA, sc-304) and mouse anti-Nedd4-1 (BD-Biosciences) antibodies for 1 hour at RT. The cells were then washed with PBS and labeled with Alexa Fluor −488 and −568 secondary antibodies (Molecular Probes, Eugene, OR, USA) and Hoechst nuclear stain in PBS for 1 hour. Samples were washed with PBS, mounted and stored at 4°C until imaged.

Cellular fluorescence was visualized with an Olympus BX50 microscope (Olympus America, Center Valley, PA, USA) using 40× magnification. Images were captured using CellSens Software (Olympus America). TRITC and FITC filter sets were used to visualize Nedd4-1 and MyoD expressing cells, respectively.

### Statistical Analyses

Continuous data are reported as a mean and standard error. Two way ANOVA was used to compare normalized measures (muscle weights, fibre CSA, protein levels) between *Nedd4-1* SMS-KO and *myo^Cre^;Nedd4-1^+/+^* control mice at 1 and 2 weeks post denervation. Differences in absolute measures (muscle weights or fibre CSA) between *Nedd4-1* SMS-KO and *myo^Cre^;Nedd4-1^+/+^* control innervated and denervated gastrocnemius at 1 week or 2 weeks were assessed by one way ANOVA followed by post-hoc Bonferroni's multiple comparison tests. Statistical significance was assumed in all cases if *p<*0.05.

## Results

### Generation of *Nedd4-1 SMS-KO* mice

**Figure 1:**
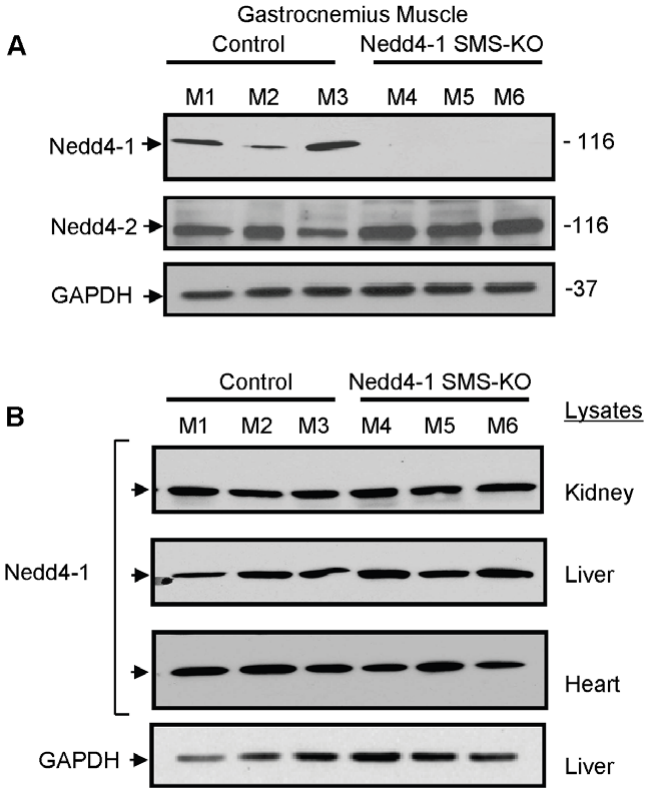
Nedd4-1 protein is absent from *Myo^Cre^;Nedd4-1^flox/flox^* (*Nedd4-1* SMS-KO) skeletal muscle. (A) SDS-PAGE and Nedd4-1 Western blotting (WB) of 50 µg of gastrocnemius muscle lysates from representative *Nedd4-1* SMS-KO mice (M4, M5, M6) and littermate *Myo^Cre^;Nedd4-1^+/+^* control mice (M1, M2, M3) reveal the absence of Nedd4-1 in the *Nedd4-1* SMS-KO muscle. Nedd4-2 protein remains equally expressed in the muscle of *Nedd4-1* SMS-KO and *Myo^Cre^;Nedd4-1^+/+^* control mice. GAPDH served as a loading control. (B) WB of heart, liver and kidney protein lysates reveal equal Nedd4-1 protein in these tissues in the *Nedd4-1* SMS-KO and littermate *Myo^Cre^;Nedd4-1^+/+^* control mice. GAPDH served as a loading control for all blots; liver lysate is shown as a representative blot.

To confirm deletion of Nedd4-1 protein in the *Nedd4-1* SMS-KO skeletal muscle, we performed SDS-PAGE and Nedd4-1 Western blotting (WB) analysis of gastrocnemius muscle lysates from *Nedd4-1* SMS-KO and littermate *Myo^Cre^;Nedd4-1^+/+^* control mice (Figure 1). The results reveal the absence of Nedd4-1 protein in the *Nedd4-1* SMS-KO muscle while a GAPDH loading control remains unaltered (Figure 1A). When other tissues (heart, liver and kidney) were examined, the amount of Nedd4-1 protein in *Nedd4-1* SMS-KO and littermate control mice was equivalent in all tissues (Figure 1B), demonstrating the skeletal muscle specificity of our *Nedd4-1* knockout. Furthermore, to ensure that the knock-out was specific for Nedd4-1, expression of Nedd4-2 was confirmed in the *Nedd4-1* SMS-KO skeletal muscle (Figure 1A). These data validate our genetic model and confirm the specificity of the Nedd4-1 null phenotype in skeletal muscle.

### Denervation increases Nedd4-1 protein expression

**Figure 2:**
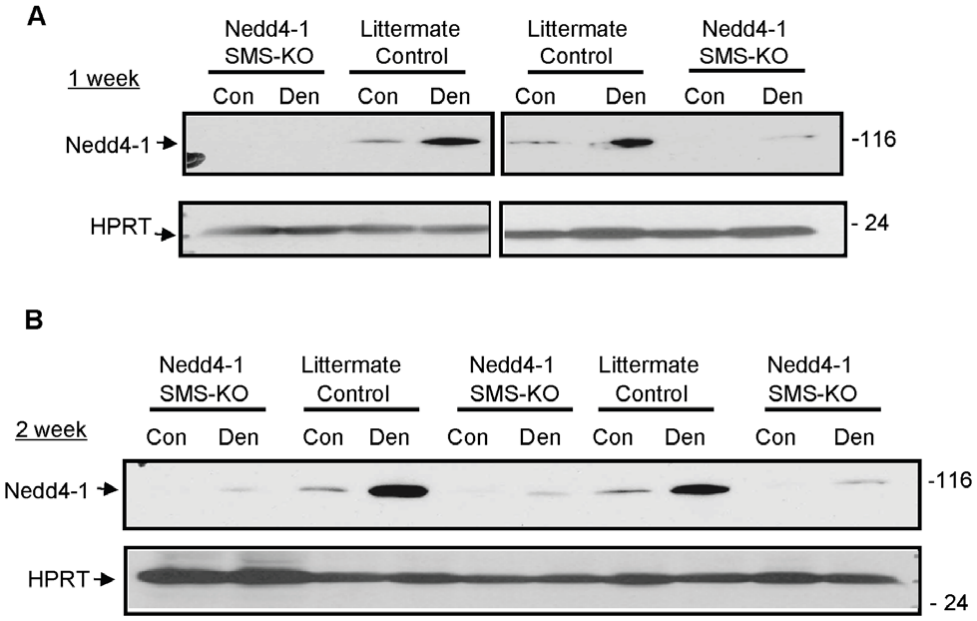
Denervation induces increased Nedd4-1 protein expression in gastrocnemius muscle. *Nedd4-1* SMS-KO and littermate *Myo^Cre^;Nedd4-1^+/+^* control mice underwent right tibial nerve transection with the left hind limb serving as control. Western blotting (WB) of 50 µg gastrocnemius protein lysates denervated for 1 week (A) and 2 weeks (B) show increased Nedd4-1 protein in the denervated (Den) compared to the contralateral control (Con) muscle of *Myo^Cre^;Nedd4-1^+/+^* mice. Nedd4-1 is absent from the *Nedd4-1* SMS-KO control gastrocnemius, but trace Nedd4-1 becomes evident with denervation. Representative blots are shown (n = 8 to 9 mice/cohort).

Previously we, and others, have shown increased Nedd4-1 expression in gastrocnemius muscles undergoing denervation or unloading induced atrophy [15], [16], [30]. To confirm this phenomenon in our genetic mouse model, *Nedd4-1* SMS-KO and littermate *Myo^Cre^;Nedd4-1^+/+^* control mice underwent right tibial nerve transection with the left hind limb serving as control. Gastrocnemius muscles were harvested 1 and 2 weeks post-denervation, weighed and lysed for Western Blotting (Figure 2A, 2B). Nedd4-1 expression in the denervated compared to the contralateral control muscle of the control mice was increased dramatically. Nedd4-1 is absent from the *Nedd4-1* SMS-KO control gastrocnemius (as noted above, Figure 1), but trace Nedd4-1 expression becomes evident with denervation.

### Trace Nedd4-1 expression in denervated gastrocnemius muscle of *Nedd4-1* SMS-KO results from satellite cell/myoblast proliferation

**Figure 3:**
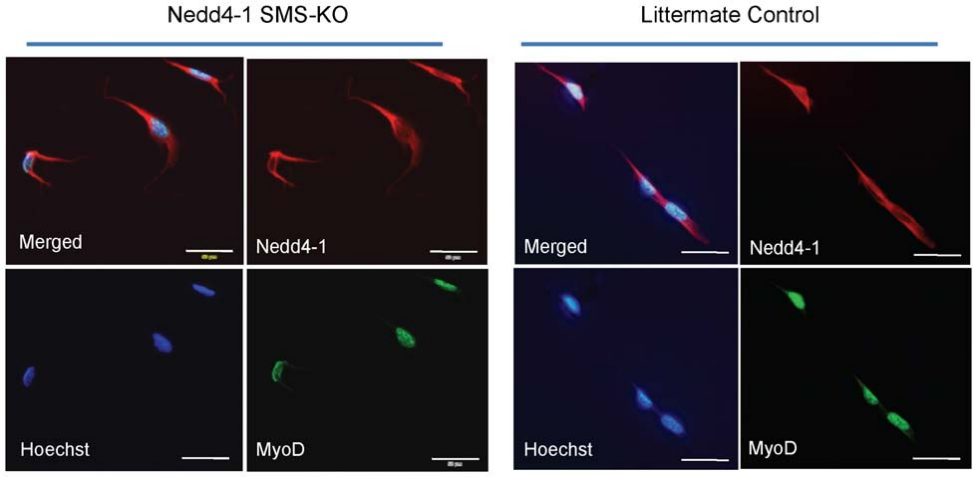
Satellite cells/myoblasts isolated from *Nedd4-1* SMS-KO mice express Nedd4-1. Muscle satellite cells/myoblasts were isolated from *Nedd4-1* SMS-KO mice and littermate *Myo^Cre^;Nedd4-1^+/+^* control mice and maintained in culture. Cultures were immunostained for Nedd4-1 (red) and MyoD (green) and nuclei were visualized with Hoechst (blue). MyoD, a specific marker of myoblasts, is expressed in nuclei; Nedd4-1 is predominantly cytoplasmic. Myoblasts derived from both *Nedd4-1* SMS-KO and littermate control mice demonstrate co-expression of MyoD and Nedd4-1. Immunostaining with 2° antibodies alone served as a negative control and did not demonstrate non-specific staining (data not shown). Scale bar shown = 50 µm.

**Figure 4:**
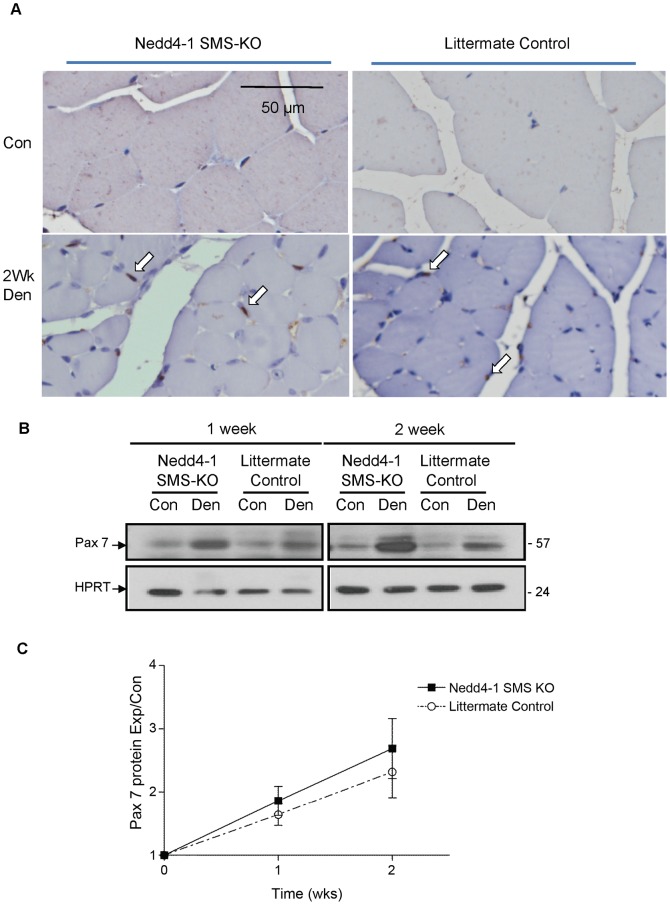
Satellite cell/myoblast population is increased in denervated gastrocnemius muscle. (A) Cross sections of gastrocnemius muscle from *Nedd4-1* SMS-KO and littermate *Myo^Cre^;Nedd4-1^+/+^* mice were immunostained for the satellite cell/myoblast marker Pax 7 (brown nuclei, indicated by white arrows). Myonuclei are counterstained purple with hematoxylin. There is an apparent increase in satellite cell number in the denervated gastrocnemius of both *Nedd4-1* SMS-KO and littermate *Myo^Cre^;Nedd4-1^+/+^* control mice. (B) Similarly, representative Western blots (WB) of gastrocnemius protein lysates denervated for 1 week (left panel) and 2 weeks (right panel) show increased expression of Pax 7 in the denervated (Den) compared to the contralateral control (Con) muscle of *Nedd4-1* SMS-KO and littermate *Myo^Cre^;Nedd4-1^+/+^* mice. HPRT served as loading control. (C) The chemiluminescent signal was quantified and Pax-7 protein levels were normalized to corresponding HPRT levels (lane matched). Normalized Pax-7 levels in denervated (Exp) muscle were expressed as a fraction of the normalized Pax-7 level in control (Con) muscle and these values are depicted in the graph. Pax-7 is significantly increased in the denervated gastrocnemius muscle of both *Nedd4-1* SMS-KO and *Myo^Cre^;Nedd4-1^+/+^* mice at both 1 and 2 weeks post-tibial nerve transection (n = 6 mice/group, *p*<0.05), but there is no difference in the magnitude of the increase between *Nedd4-1* SMS-KO and *Myo^Cre^;Nedd4-1^+/+^* mice. Data are presented as the mean ± SEM.

To determine the source of the trace Nedd4-1 expression in the denervated gastrocnemius muscle, we assessed muscle satellite cells/myoblasts for Nedd4-1 protein expression. Quiescent satellite cells/myoblasts initiate proliferation in a reparative response to denervation injury, increasing from about 1%, to up to 10%, of all muscle cells [31]. Our knockout design does not delete Nedd4-1 from skeletal muscle satellite cells/myoblasts. During embryogenesis, expression of the transcription factors MyoD and Myf5 specify mesodermal precursor cells to the myogenic lineage, generating proliferative myoblasts [32]. Subsequent expression of myogenin and MRF4 induce terminal differentiation of myoblasts to mononucleated myocytes, which then fuse together to form multinucleated muscle fibres. During skeletal muscle development, a subpopulation of myoblasts, known as satellite cells, fail to differentiate but remain associated with the myofibres and retain the capacity to proliferate and differentiate so as to regenerate skeletal muscle post-natally [32]. Since Cre-recombinase expression in our mice is under the control of the myogenin promoter, Nedd4-1 should still be expressed post-natally in the quiescent satellite cells and proliferating myoblasts of Nedd4-1 SMS-KO mice, because myogenin is not yet expressed in these progenitor cells. Indeed, we were able to demonstrate the co-expression of Nedd4-1 and myoD in cultured myoblasts derived from the *Nedd4-1* SMS-KO mice (Figure 3). We additionally demonstrate increased expression of the satellite cell/myoblast marker Pax-7 [32] in the denervated gastrocnemius (Figures 4A, 4B and 4C) of both *Nedd4-1* SMS-KO and littermate control mice, in keeping with the known activation of satellite cell/myoblast proliferation following denervation [33], [34]. These data together support the conclusion that trace Nedd4-1 expression induced in the denervated gastrocnemius of *Nedd4-1* SMS-KO mice likely results from associated satellite cell/myoblast proliferation.

### *Nedd4-1* SMS-KO mice are partially protected against denervation atrophy

As a measure of muscle sparing, denervated and control innervated gastrocnemius muscles were harvested 1 and 2 weeks post nerve transection, weighed, fixed, cut on cross-section and immunostained for fast twitch myosin to identify type I and type II fibres.

**Figure 5:**
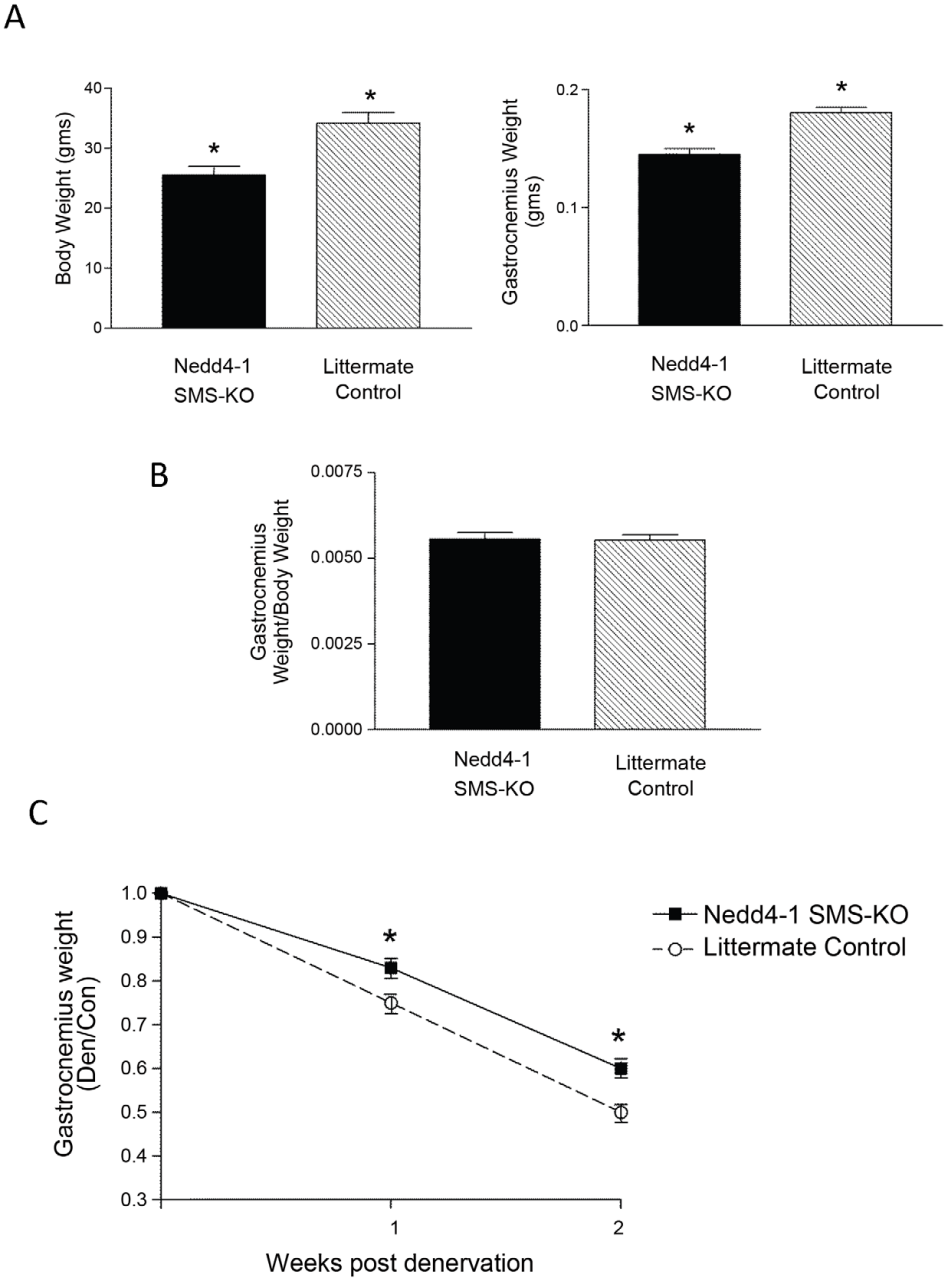
*Nedd4-1* SMS-KO gastrocnemius muscle weights are partially spared from denervation induced atrophy. (A) Total body weights (left panel) and gastrocnemius muscle weights (right panel) of *Nedd4-1* SMS-KO and littermate *Myo^Cre^;Nedd4-1^+/+^* control mice. *Nedd4-1* SMS-KO mice are slightly smaller, and their gastrocnemius muscle weigh less, than littermate controls (n = 16 pairs, * p<0.05). (B) There is no difference in the basal gastrocnemius muscle mass between the *Nedd4-1* SMS-KO and control mice when muscle weight is normalized to body weight p>0.05). (C) *Nedd4-1* SMS-KO and littermate control mice underwent right tibial nerve transection, denervating the gastrocnemius muscle, with the intact left hind limb serving as internal control. Denervated gastrocnemius muscle weights are normalized to the weight of the contralateral control muscle. Denervated muscle of *Nedd4-1* SMS-KO (solid line, closed squares) mice demonstrates an attenuated atrophic response weighing significantly more than in *Myo^Cre^;Nedd4-1^+/+^* (dashed line, open circles) mice at 1 and 2 weeks post denervation, (*p<0.05, n = 8 or 9 mice/cohort. Data are presented as the mean ± SEM).

At baseline, the *Nedd4-1* SMS-KO mice are slightly smaller than, and the gastrocnemius muscle weighs less than, that of littermate control mice (Figure 5A). However, there is no difference in the muscle mass when normalized to body weight (Figure 5B).

**Figure 6:**
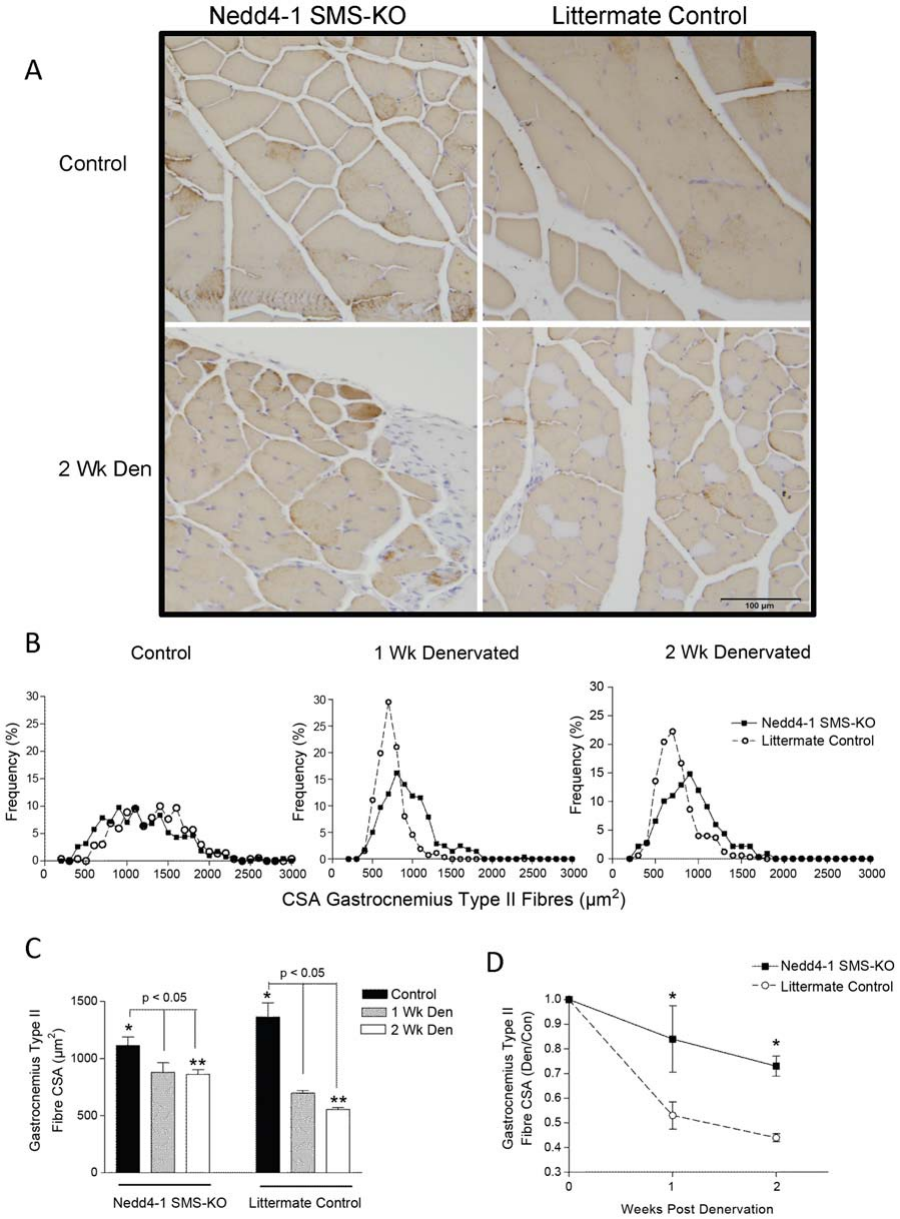
Nedd4-1 deletion attenuates denervation induced decreases in gastrocnemius Type II fibre cross-sectional area. (A) Cross sections of denervated and control gastrocnemius muscle of *Nedd4-1* SMS-KO and *Myo^Cre^;Nedd4-1^+/+^* (littermate control) mice were immunostained with an anti-fast twitch myosin antibody and counterstained with hematoxylin. Fast twitch type II fibres stain brown, and slow twitch type I fibres stain light purple. Representative cross sections from control and 2 week denervated gastrocnemius muscle from both *Nedd4-1* SMS-KO and littermate control mice are shown. (B) Histograms of type II fibre cross sectional areas (CSA) for control innervated (left panel), 1 week denervated (middle panel) and 2 week denervated (right panel) gastrocnemius muscles in *Nedd4-1* SMS-KO (solid line, closed squares) and littermate control (dashed line, open circles) mice are shown. (C) At baseline, *Nedd4-1* SMS-KO gastrocnemius type II fibre CSA is smaller than that of *Myo^Cre^;Nedd4-1^+/+^* mice (*). Following denervation, there is a significant decrease in type II fibre CSA for both cohorts of mice. However the CSA of *Nedd4-1* SMS-KO muscle fibres denervated for 2 weeks is significantly larger than fibres of *Myo^Cre^;Nedd4-1^+/+^* mice (**) denervated for 2 weeks. (D) Similarly, when Type II fibre CSA in denervated gastrocnemius muscle is normalized to CSA in the contralateral control muscle, type II fibres are significantly larger in *Nedd4-1* SMS-KO (solid line, closed squares) mice compared to *Myo^Cre^;Nedd4-1^+/+^* (dashed line, open circles) mice at both 1 week and 2 weeks post denervation. A minimum of 300 myofibres/muscle was measured, (** and *p<0.05, n = 8 or 9 mice/cohort. Data are presented as the mean ± SEM).

Denervated gastrocnemius muscle weights, normalized to the weight of the contralateral limb control muscle, were significantly greater in *Nedd4-1* SMS-KO compared to littermate *Myo^Cre^;Nedd4-1^+/+^* mice at both 1 and 2 weeks post tibial nerve transection (Figure 5C), demonstrating an attenuated atrophic response in the Nedd4-1 null muscle. Similarly, the type II fibre CSA was significantly larger in the denervated gastrocnemius muscle of *Nedd4-1* SMS-KO compared to control *myo^Cre^;Nedd4-1^+/+^* mice (Figure 6). This is despite the fact that control innervated *myo^Cre^;Nedd4-1^+/+^* gastrocnemius type II fibre CSA was larger than that of *Nedd4-1* SMS-KO mice (Figure 6B and 6C). Denervation induces a fibre type switch from type I to type II fibres, and as a result an inadequate number of type I fibres was available for measurement in the formalin fixed portions of denervated gastrocnemius.

### Levels of the Nedd4-1 substrates MTMR4, Notch-1 and FGFR1, are not altered by loss of Nedd4-1 in muscles

To identify potential Nedd4-1 muscle substrates that regulate the loss of muscle mass we focused on known Nedd4-1 substrates that have been reported to demonstrate decreased protein levels in denervated and/or unloaded or immobilized muscle, and identified potentially three; MTMR4, FGFR1 and Notch-1 [16], [35], [36]. We reasoned that if Nedd4-1 signalled downstream via either MTMR4, FGFR1 or Notch-1 to induce muscle atrophy, then these protein(s) would be protected from degradation in the denervated gastrocnemius muscle of *Nedd4-1* SMS KO mice.

**Figure 7:**
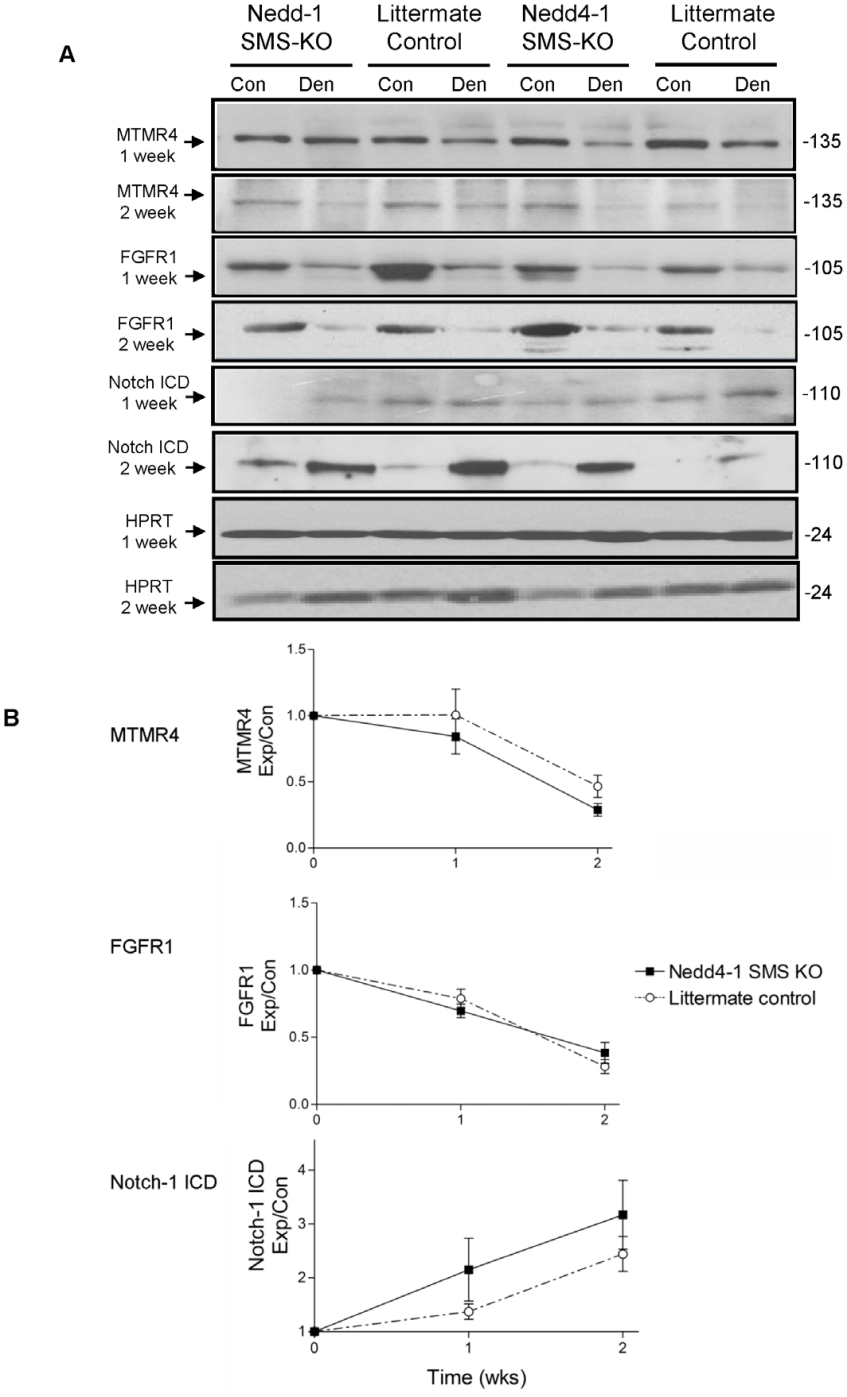
Levels of MTMR4 and FGFR-1 are not maintained in the denervated gastrocnemius of *Nedd4-1* SMS-KO mice, while cleaved Notch-1 expression is increased. (A) Representative Western blots of MTMR4, FGFR1, cleaved Notch-1 (Notch-1 ICD) and HPRT (as a loading control) in protein lysates from denervated gastrocnemius muscle (Den) or the contralateral control gastrocnemius (Con) muscle in *Nedd4-1* SMS-KO and littermate *Myo^Cre^;Nedd4-1^+/+^* control mice at 1 and 2 weeks post tibial nerve transection. (B) The chemiluminescent signal was quantified, MTMR4, FGFR1 and Notch-1 protein levels were normalized to the corresponding HPRT levels (lane matched) and numerical values are depicted in bar graphs. For each of MTMR4, FGFR1 and Notch-1, protein levels in denervated (Exp) muscle are expressed as a fraction of the protein level in control (Con) muscle. MTMR4 levels were significantly decreased in denervated compared to control gastrocnemius muscle in both *Nedd4-1* SMS-KO (n = 6) and *Myo^Cre^;Nedd4-1^+/+^* mice (n = 6) mice at 2 weeks (p<0.05), but there was no difference in the magnitude of MTMR4 decrease between the *Nedd4-1* SMS-KO compared to control mice. FGFR1 levels were similarly significantly decreased in denervated compared to control gastrocnemius muscle in both *Nedd4-1* SMS-KO (n = 8) and littermate *Myo^Cre^;Nedd4-1^+/+^* control mice (n = 8) mice at 1 week and 2 weeks (p<0.05) post tibial nerve transection, and again there was no difference in the magnitude of the decrease between the 2 cohorts of mice at either timepoint. Cleaved Notch-1 levels (Notch ICD) were significantly increased in denervated compared to control gastrocnemius in both *Nedd4-1* SMS-KO (n = 6) and *Myo^Cre^;Nedd4-1^+/+^* control mice (n = 6) mice at 2 weeks (p<0.05), but not at 1 week. There was no difference in the magnitude of the increase between the 2 cohorts of mice. Data are mean ± SEM.

We found that levels of both MTMR4 and FGFR1 proteins decreased in the denervated gastrocnemius muscle of control mice coincident to the increase in Nedd4-1 (Figures 7A and 7B), but there was no attenuation of that decrease for either protein in the denervated gastrocnemius of *Nedd4-1* SMS-KO mice, suggesting that neither MTMR4 nor FGFR1 are substrates involved in Nedd4-1's mediation of denervation induced muscle atrophy. In contrast, cleaved Notch-1 demonstrated an increase in the 2 week denervated gastrocnemius compared to control muscle (Figures 7A and 7B) and the extent of this increase was similar between *Nedd4-1* SMS-KO and littermate control mice, again suggesting that Notch-1 does not serve as a substrate in Nedd4-1's induction of skeletal muscle atrophy. Notch-1 full length (FL) was not detectable in muscle lysates with several Notch-1 antibodies tested (data not shown).

## Discussion

In the present study we generated a novel genetic tool, the *Nedd4-1* SMS-KO mouse, to assess the role of Nedd4-1 in the loss of skeletal muscle mass. We subjected the mice to a well-validated model of denervation-induced skeletal muscle atrophy. We showed heavier weights and larger type II fibre cross sectional area in denervated relative to control gastrocnemius muscle of the *Nedd4-1* SMS-KO mice, demonstrating that the ubiquitin ligase Nedd4-1 participates in the development of denervation-induced skeletal muscle atrophy.

Others have also interrogated the role of Nedd4-1 in muscle proteolysis and reported that acute over-expression of recombinant Nedd4-1 via cDNA electrotransfer into muscle did not induce myofibre atrophy at seven days in weight bearing rats, nor did over-expression of a catalytically inactive dominant negative Nedd4-1 rescue myofibres from atrophy induced by seven days of unloading [16]. The reason for these findings, which are contradictory to our observations, is not clear. We speculate they may result from the use of a dominant negative, as opposed to a genetic or knockdown approach and/or inadequate levels or duration of Nedd4-1 expression *in vivo*. With exogenous expression, the early and single seven day time point of assessment may have been inadequate to observe the Nedd4-1 effect, which remains up-regulated in muscle for many weeks following denervation [15]. The generation of our genetic mouse model here has provided us with a unique tool with which to bypass these limitations and demonstrate a positive role for Nedd4-1 in the mediation of denervation induced skeletal muscle atrophy.

The contribution of Nedd4-1 to the regulation of denervation induced muscle atrophy appears to be smaller than that provided by either atrogin-1 or MuRF1. Genetic deletion of either of these ligases under similar conditions resulted in the retention of a larger muscle mass at fourteen days post denervation, when compared to our results here with Nedd4-1 [13]. The influence of Nedd4-1 on muscle may be more extensive than what we demonstrate at this time, given that Nedd4-1 expression is retained in satellite cells/myoblasts in our *Nedd4-1* SMS KO mice. Deletion of Nedd4-1 from the satellite cell population may enhance the satellite cell/myoblast proliferative response to denervation, thus further inhibiting the development of atrophy, but we were unable to assess this phenomenon in our genetic model. Nonetheless, given that the individual absence of each of Nedd4-1, atrogin-1 or MuRF1 was insufficient to provide complete protection against muscle atrophy, it is likely that the co-ordinated activity of these three ligases together with other proteolytic systems is necessary to mediate the loss of muscle mass following denervation.

We found the deletion of Nedd4-1 from skeletal muscle provided protection from denervation induced atrophy early post denervation (seven days), like atrogin-1 and in contrast to MuRF1 which demonstrates a delayed protective effect, being evident at only fourteen days post denervation [13]. The varied temporal effects of these E3 ligases may result from the different downstream substrates that each engages. MuRF1 ubiquitinates several muscle contractile/structural proteins, resulting in proteolysis of primarily thick filament fibres [37]–[39]. In contrast, it appears that atrogin-1 mediates skeletal muscle atrophy by ubiquitinating proteins that stimulate protein synthesis and muscle precursor cell proliferation and differentiation, the eukaryotic initiation factor eIF3-f and MyoD respectively, marking them for proteasome-mediated proteolysis [40], [41]. To identify the substrates that Nedd4-1 targets to promote the induction of denervation induced skeletal muscle atrophy we focused on known Nedd4-1 substrates that demonstrate decreased levels in denervated and/or unloaded or immobilized muscle; MTMR4, which we identified previously [35], FGFR1 [42] and Notch-1 [16].

MTMR4 belongs to a family of dual specificity phosphatases, the myotubularins, which regulate phosphoinositol metabolism in early endosomes, thereby regulating endocytosis and trafficking of proteins, including growth factor receptors [43]–[46]. Knockout of the related MTM1 results in a progressive atrophic myopathy starting at a few weeks after birth, suggesting MTM1 is required for muscle structural maintenance [47]. Ectopic over-expression of FGFR1 in mouse gastrocnemius by electroporation significantly inhibited inactivity-induced atrophy, and FGFR1 is increased in myofibres that are partially resistant to inactivity-induced atrophy [36]. While we observed decreased MTMR4 and FGFR1 protein levels in the denervated gastrocnemius muscle of control mice coincident with the increase in Nedd4-1, in keeping with the possibility that Nedd4-1 mediated ubiquitination of these target proteins is causally associated with the loss of muscle mass, this does not appear to be the case as we found no attenuation of this decrease in MTMR4 and FGFR1 protein levels in the denervated gastrocnemius of *Nedd4-1* SMS-KO mice.

Notch-1 is a transmembrane receptor protein that is sequentially cleaved upon ligand binding, releasing its “activated” intracellular domain, which translocates from the cytosol to the nucleus to influence transcription [48]. In muscle satellite cells Notch-1 positively regulates proliferation, and decreased expression of Notch-1 coincides with the onset of terminal differentiation to myocytes [29], [49]. A role for Notch-1 in mature muscle fibres has not yet been elucidated.

The response of Notch-1 to denervation has been disputed. One study demonstrates both denervation and unloading decrease Notch-1 (full length and cleaved) protein levels in muscle seven days post intervention [16], while another recent study reports an increase in cleaved Notch-1 protein in muscle, seven and 35 days following denervation injury [50]. Our own work here similarly shows that Notch-1 is increased in denervated muscle, but there was no difference in the magnitude of the cleaved Notch-1 increase between *Nedd4-1* SMS-KO and littermate control mice. This increase in Notch-1 may reflect an increase in satellite cell proliferation. While denervation of skeletal muscle results in myofibre proteolysis, the muscle concurrently mounts a reparative response. Within 48 hours of denervation, satellite cells/myoblasts begin to activate, proliferate and then differentiate to myocytes that fuse to form myotubes, or bridge myofibres, in an attempt at regeneration [33], [34], [51]. With prolonged denervation (several weeks to months in rats), the satellite cell regenerative response eventually becomes exhausted [51], [52]. At both one and two weeks post tibial nerve transection, we demonstrated a marked increase in the satellite cell/myoblast marker Pax-7 [32] in the denervated gastrocnemius muscle of both *Nedd4-1* SMS-KO and littermate control mice, in keeping with the satellite cell proliferative response to denervation. Thus, we currently find no evidence of Nedd4-1 engaging Notch-1 to mediate denervation induced skeletal muscle atrophy. The downstream mechanism by which Nedd4-1 contributes to the induction of denervation induced skeletal muscle atrophy currently remains unknown.

In conclusion, using a novel and unique genetic tool, the *Nedd4-1* SMS-KO mouse, we have demonstrated that the ubiquitin ligase Nedd4-1 participates in the positive regulation of denervation induced skeletal muscle atrophy.
